# BMP Signaling Maintains Healthy Joint Cartilage

**DOI:** 10.1371/journal.pbio.0020395

**Published:** 2004-10-19

The alarm clock rings and you jump straight out of bed, rattling downstairs to start the day. Or maybe you creak downstairs, each step a struggle because of stiffness and pain in your knees and other joints. If the second description fits the start of your day, then maybe, like 70 million Americans, you have arthritis, one of the most prevalent chronic health problems in the United States.

Arthritis is an umbrella term for more than 100 medical conditions. What all forms of arthritis have in common is that they affect our joints-places where two or more bones meet. In healthy joints, the ends of the bones are covered with cartilage, a tough but smooth tissue that, like the oil in a car engine, reduces friction between the moving parts. In the most common form of arthritis-osteoarthritis-breakdown of this cartilage, which is called articular cartilage, means the bones rub together, causing pain and loss of movement. Risk factors for osteoarthritis include age and family history.

If we could understand the molecular mechanisms that create and maintain articular cartilage, it might be possible to discover what goes wrong in our joints as we age and to find better treatments for arthritis. Embryologists have already discovered quite a bit about the earliest stages of joint formation. It is known, for example, that stripes of cells that form between developing bones subsequently develop into the permanent cartilage found in joints. Several members of a family of secreted proteins known as bone morphogenetic proteins (BMPs) are expressed in these stripes of cells, implicating BMP signaling (the transmission of messages produced by BMPs binding to cell-surface receptors) in early joint development.

**Figure:**
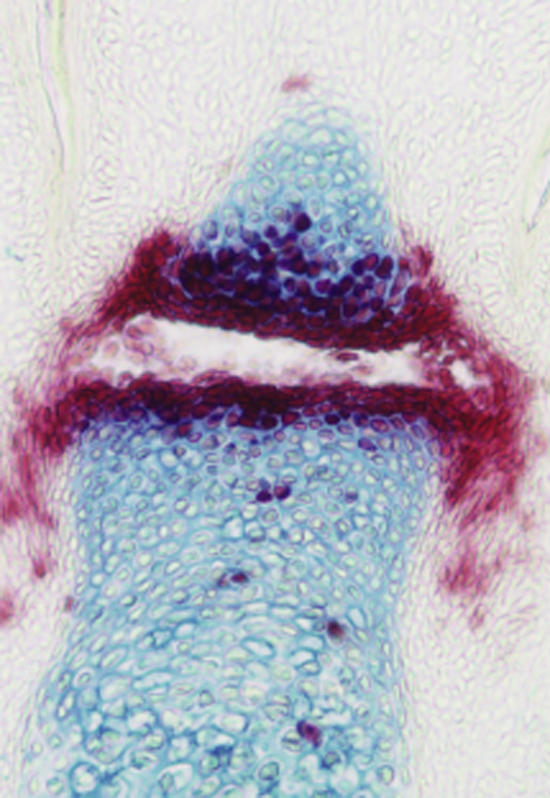
Targeting genes in joints

David Kingsley's team has been investigating whether BMPs are also involved in the later development and maintenance of joint cartilage. To do this, the researchers designed a genetic system that inactivates BMP signaling late in mouse embryonic development. They inserted special DNA sequences called *loxP* sites on either side of *Bmpr1a*, a gene that encodes one of the BMP receptors. The *loxP* sites have no effect until an enzyme known as Cre is expressed, and then the DNA between the *loxP* sites is cut out and discarded. Because Kingsley's team knew that global inactivation of *Bmpr1a* early in development causes embryonic death, they linked the gene for Cre to DNA sequences that limit its expression to those regions of the embryo where joints eventually develop. The result: a mouse strain in which *Bmpr1a* receptor function is specifically lost only in tissues destined to become joints.

Most of the joints in this mouse strain formed normally. However, the mice rapidly developed severe arthritis after birth. By 7 days old, the expression of proteins normally found in cartilage was reduced, although at this stage the knee, for example, looked normal. By 7 weeks old (adulthood for mice), there were clear structural changes in the knee joints, and the articular cartilage was thinner and showed signs of wearing away. By 9 months old, the knees of the mutant mice largely lacked articular cartilage and the unprotected leg bones seemed to rub directly against each other.

All told, the joints in these mutant mice closely resembled those in people with osteoarthritis, suggesting that BMP signaling is necessary for the maintenance of healthy articular cartilage. This raises the possibility that mutations in BMP signaling components may underlie some of the genetic variation in human osteoarthritis risk and suggests that treatments designed to mimic or augment BMP signaling might help to maintain healthy joints. Finally, the genetic system described by Kingsley and coworkers should be useful for future investigations into joint formation and maintenance.

